# The efficacy of resveratrol in controlling hypertension: study protocol for a randomized, crossover, double-blinded, placebo-controlled trial

**DOI:** 10.1186/s13063-016-1426-x

**Published:** 2016-06-23

**Authors:** Ali Movahed, Afshin Ostovar, Daryoush Iranpour, Sijo Joseph Thandapilly, Pema Raj, Xavier Lieben Louis, James Michael Smoliga, Thomas Netticadan

**Affiliations:** The Persian Gulf Tropical Medicine Research Center, Biochemistry Group, Bushehr University of Medical Sciences, Bushehr, Iran; Canadian Centre for Agri-Food Research in Health and Medicine, Winnipeg, R2H 2A6 Canada; Department of Cardiology, Faculty of Medicine, Bushehr University of Medical Sciences, Bushehr, Iran; Agriculture and Agri-Food Canada, Winnipeg, Manitoba R3T 2M9 Canada; Department of Physiology and Pathophysiology, University of Manitoba, Winnipeg, Manitoba R3E 0J9 Canada; Department of Physical Therapy, High Point University, High Point, NC 27262 USA; Department of Basic Pharmaceutical Sciences, High Point University, High Point, NC 27262 USA; Heart Failure Research Laboratory, Canadian Centre for Agri-Food Research in Health and Medicine, R2035, St. Boniface Research Centre, 351 Tache Avenue, Winnipeg, Manitoba R2H 2A6 Canada

**Keywords:** Resveratrol, Hypertension, Blood pressure, Polyphenol

## Abstract

**Background:**

Hypertension is a global health concern for which novel treatment strategies are necessary. The aim of this study is to evaluate the efficacy of resveratrol (trans-3, 5, 4′-trihydroxystilbene, a polyphenol present in grapes) in controlling blood pressure in participants diagnosed with prehypertension and stage 1 hypertension.

**Methods/design:**

In a randomized, crossover, double-blinded, placebo-controlled study, 50 participants with prehypertension (diastolic blood pressure and systolic blood pressure, 80–89 mmHg and 120–139 mmHg, respectively) and 50 participants with stage 1 hypertension (diastolic and systolic, 90–99 mmHg and 140–159 mmHg, respectively) will be assigned to receive resveratrol (99 % pure, from Biotivia Longevity Bioceuticals LLC Company, USA, in 500 mg capsules, twice daily for 4 weeks, orally) or placebo (500 mg neutral microcellulose capsules, twice daily for 4 weeks) in a 2 × 2 crossover design (4 weeks treatment—4 weeks washout—4 weeks treatment). The blood pressure of each participant will be recorded (a mean of two times within a 15-minute interval) every week during the study. The participants in the prehypertensive group will not receive any medication, while those in the stage 1 hypertensive group will continue to receive their routine medications during the study. Blood samples will be taken from all groups and examined for various biochemical parameters.

**Discussion:**

This trial will help to establish whether resveratrol is an effective antihypertensive agent in prehypertensive and stage 1-hypertensive patients. The trial outcome will provide novel insight into the clinical efficacy of resveratrol and provide valuable information for conducting future clinical studies with resveratrol.

**Trial registration:**

Iranian Registry of Clinical Trials, IRCT201407078129N7. Registered on 15 August 2014.

## Background

Worldwide, one in three adults is afflicted with elevated blood pressure (BP) or hypertension; in comparison, diabetes affects one in ten adults [1]. High BP is one of the most common risk factors for cardiovascular disease, affecting approximately 40 % of the adult population worldwide [2]. Currently, it accounts for 45 % and 51 % of deaths resulting from coronary artery disease and stroke, respectively. In addition, its prevalence is projected to reach as high as 1.5 billion by 2025 [2]. Elevated BP results from varying factors such as genetics, diet, lifestyle, and combinations thereof [3]. Hypertension may also further increase morbidity and mortality due to other diseases in the presence of other risk factors. Strategies for the prevention and management of hypertension and associated adverse consequences are based on lifestyle modification as well as pharmacological interventions [3]. Notably, clinical trial evidence showed that a large number of hypertensive participants (20–30 %) are resistant to the maximum tolerated dose of antihypertensive drugs administered in various combinations, and thus, managing hypertension remains an enormous challenge [4]. Accordingly, more effective alternative treatment options that can clinically resolve this debilitating condition should be developed to improve patient outcomes and reduce the public health burden.

Wide-ranging dietary modifications and interventions that include functional foods and nutraceuticals have been shown to have promising antihypertensive effects in the preclinical and clinical studies [3, 5–7]. The combination of lifestyle, pharmacological, and other alternative approaches may prove beneficial in individuals affected by hypertension and may also reduce the risk of the previously mentioned diseases.

In the past decade, considerable interest has developed for using the plant polyphenol resveratrol to combat hypertension and other forms of cardiovascular and metabolic diseases. Resveratrol has been reported to possess a dose-dependent antihypertensive effect in various animal models of systemic hypertension such as the spontaneously hypertensive rats (SHR) model, the angiotensin (Ang) II-infused mouse model, the two-kidney one-clip hypertensive rat, and in partially nephrectomized rats [8]. Moreover, resveratrol at a low dose (2.5 mg/kg body weight/day) has been shown to enhance the effect of other antihypertensive medication in the SHR [9]. Additionally, resveratrol protects against and/or reverses pulmonary hypertension in rat [10, 11]. Resveratrol is also effective in preventing high-fat-induced and high-sucrose-induced arterial stiffness in nonhuman primates [12]. Resveratrol-mediated reduction in hypertension has been attributed to various mechanisms, including improvement in oxidative stress, inflammation, endothelial dysfunction, and vasodilation [8]. However, clinical data from well-designed trials, which can be translated to humans as a treatment option, are scarce. Notably, resveratrol has been reported to lower BP in diabetic patients [6, 13, 14]; however, no study to date has specifically examined its potential in lowering BP in persons diagnosed with hypertension.

We hypothesize that resveratrol administration (1 g/day for 4 weeks) alone will reduce the BP or will complement the standard antihypertensive medication in hypertensive patients. For example, resveratrol may also have an incremental effect in lowering high BP when taken along with the standard antihypertensive medication. This would help to reduce the dosage of antihypertensive medications that have side effects. To test our hypothesis, a crossover, randomized, double-blinded, placebo-controlled clinical trial involving 50 participants with prehypertension and 50 participants with stage 1 hypertension was designed. Blood pressure and biochemical parameters will be analyzed to test the antihypertensive efficacy and mechanisms of action of resveratrol in the study subjects.

## Methods/design

### Design overview and ethics approval

**Fig. 1:**
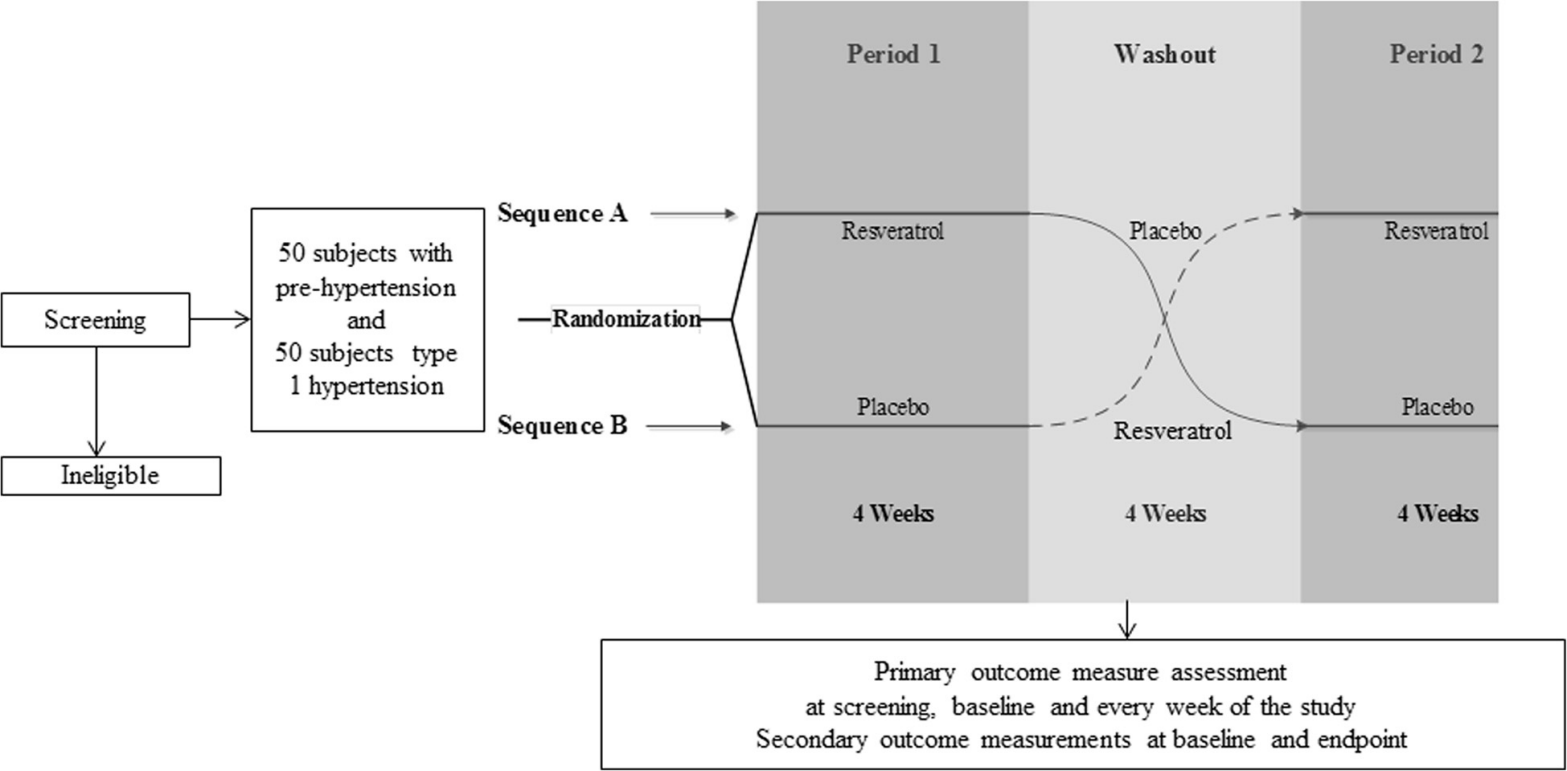
Trial design

This study is a crossover, randomized, double-blinded, placebo-controlled, single-center trial with an allocation ratio of 1:1 (Fig. 1). The participants, physician (Daryoush Iranpour), principal investigator and physician (Ali Movahed), and statistical consultant (Afshin Ostovar) will be blinded to the allocation status. The records of allocations will be kept confidential by methodology consultant and will be disclosed only after blinded statistical analyses or by request from Data and Safety Advisory Board. This trial is an investigator-initiated study sponsored by the Persian Gulf Tropical Medicine Research Center affiliated to Bushehr University of Medical Sciences, Bushehr, Iran (Grant number: 3172, 93/4/17). The study is approved by the regional research ethics committee of BPUMS, Bushehr University of Medical Sciences, approval NO: B-93-16- 4. The trial was registered with the Iranian Registry of Clinical Trials (IRCT) (NO: IRCT201407078129N7) on 15 August 2014.

### Study settings, population, and recruitment

The study will be conducted at the School of Medicine of Bushehr University of Medical Sciences, Bushehr, Iran. Prehypertensive (the mean of two measurements in a 15-minute interval; diastolic and systolic BP, 80–89 mmHg and 120–139 mmHg, respectively) and stage-1 hypertensive (the mean of two measurements in a 15-minute interval; diastolic and systolic BP, 90–99 mmHg and 140–159 mmHg, respectively) males or females, aged between 20 and 60 years will be enrolled for the trial. The patients for this study will be recruited through doctor referral at the clinic and randomized to the treatment arm after the initial screening and with their voluntary consent.

### General objective

The objective of this trial is to determine whether resveratrol (99 % pure) treatment for 4 weeks will lower BP in prehypertensive and stage 1-hypertensive patients.

### Specific objectives

#### Primary objectives

The primary objectives of this trial are to determine the BP-lowering effects of resveratrol on systolic, diastolic, and mean arterial BP in participants diagnosed with prehypertension and stage 1 hypertension.

#### Secondary objectives

The secondary objectives are as follows:

- To determine if treatment with resveratrol lowers levels of renin, angiotensin II, endothelin, norepinephrine, tumor necrosis factor-α (TNF-α), and oxidative stress markers and increases the level of nitric oxide in prehypertensive and stage 1-hypertensive patients.
- To determine the effects of resveratrol on hematologic indices in participants with prehypertension and stage 1 hypertension.
- To determine the effects of resveratrol on lipid profile in these patients.
- To determine the effects of resveratrol on liver function markers in patients with prehypertension and stage 1 hypertension.
- To determine the effects of resveratrol on renal function markers in patients with prehypertension and stage 1 hypertension.

### Specific procedures

Serum renin, angiotensin II, endothelin, norepinephrine, and TNF-α will be measured by ELISA kits (Thermo Scientific, IL, USA). Nitric oxide (NO), malondialdehyde and urinary isoprostanes will be measured by ELISA Kits, spectrophotometry, gas chromatography/mass spectrometry, respectively [15]. Hematocrit (HCT) and platelet (PLT) will be estimated using a hematology cell counter (Sysmex Analyzer). Prothrombin time (PT) and partial thromboplastin time (PTT) will be measured using a blood coagulameter. Analyses for biochemical parameters, including fasting blood glucose (FBG), will be carried out at the Persian Gulf Tropical Medicine Research Center affiliated to Bushehr University of Medical Sciences (BPUMS), Bushehr, Iran, using a Selectra 2 autoanalyzer (Vital Scientific, Spankeren, Netherlands) [16]. Serum total cholesterol and high-density lipoprotein cholesterol (HDL) will be estimated using cholesterol oxidase phenol amino antipyrine enzymatic method and triglyceride (TG) using the glycerol-3-phosphate oxidase phenol amino antipyrine enzymatic method [16]. Serum low-density lipoprotein (LDL) cholesterol will be calculated using the Friedewald formula [17]. Creatinine and blood urea nitrogen (BUN) levels will be estimated using enzymatic method. In order to measure liver function in the patients, alkaline phosphatase (ALP), gamma-glutamyl transferase (GGT), bilirubin and albumin will be measured by enzyme kinetic methods on a Selectra 2 autoanalyzer (Vital Scientific, Spankeren, Netherlands [16]).

### Eligibility criteria

Inclusion criteria are as follows:

- Prehypertensive (mean of two measurements in a 15-minute interval; diastolic and systolic BP, 80–89 mmHg and 120–139 mmHg, respectively)
- Stage 1 hypertensive (mean of two measurements in a 15 minute interval; diastolic and systolic BP, 90–99 mmHg and 140–159 mmHg, respectively)
- Male or female
- Age between 20 and 60 years
- Ability to provide informed consent
- Exclusion criteria are as follows:
- Approved or doubtful secondary hypertension
- History of chronic or acute kidney disease
- History of heart failure
- History of chronic or acute liver diseases
- History of diabetes mellitus
- History of prior cardiovascular events (acute myocardial infarction, cardiovascular diseases, percutaneous coronary angioplasty or coronary artery bypass graft)
- Pregnancy or breast feeding
- Blood arterial pressure > 180/110
- History of bowel disease of any etiology that may affect absorption/or distribution of any drug administered orally
- History of electrolyte imbalance during 3 months prior to the enrolment
- History of alcohol abuse 4 weeks prior to the enrolment
- Requiring a major surgical procedure (abdominal, thoracic, neurovascular, urological, or gynecological) during the course of the study
- Consumption of steroid hormones or nonsteroidal anti-inflammatory drugs 1 month prior to the enrolment
- History of hormonal changes (thyroid and adrenal)
- Receiving lipid-lowering drugs
- History of bleeding disorders
- Receiving blood thinners
- Regular intake of omega-3 fatty acid, vitamins, and mineral supplements
- Intention of having high intake of table salt or salty foods

### Informed consent

The patients will be informed about the trial by their doctor during clinic visit. Interested individuals will contact the study coordinator by telephone/email[JMS1] . Interested individuals will be invited to attend a first study visit by the concerned clinicians on a specified day, when a pre-screening will be conducted to exclude participants based on the inclusion/exclusion criteria[K2] . If participants show up with abnormal values for laboratory tests during pre-screening, the tests will be repeated. If tests show similar results (abnormal values) the participants will be excluded from the study. If the criteria are met, study coordinator will go through the consent form designed in local language (Persian). Finally, clinical trial details will be outlined and the participants will be given an opportunity to ask any questions/concerns. If they consent to the study, enrollment will be completed. Participants will be enrolled only with their voluntary informed consent (Appendix 1).

### Randomization and intervention

After the enrolment of the patient is completed, randomization will be done to allocate them to placebo or resveratrol arms. A stratified complete block randomization method will be used in this trial. Blocks of four will be used for this purpose. The randomization scheme will be generated using random number formulae in Microsoft Excel. Patients with prehypertension or stage 1 hypertension will be separately randomized to receive active drug (resveratrol) in 500 mg capsules, twice daily for 4 weeks (sequence A), or placebo (500 mg neutral microcellulose capsules), twice daily for 4 weeks (sequence B), in a 2 × 2 crossover design. The drug or placebo will be taken by patients at 7 to 8 a.m. in the morning on an empty stomach and at 8 to 9 p.m. with 200 cc water. The patients will be asked to record their conditions after taking the capsules in case any unusual effects are experienced. At the end of the 4 weeks, another 4-week washout period will follow, during which the patients in both sequences will receive placebo. All the participants will be followed up for an additional 1-month period for assessing any possible aftereffects (Fig. 1).

The study will be double-blinded (the patients, those who will be participating in the study and those who analyze the results will be unaware of the state of the patient with regard to receiving the active drugs or placebo). For this purpose, participants will be blinded by using a placebo that is identical to active drug in appearance, but the content is neutral cellulose. To blind those who conduct the study, the person who delivers or checks the study drug will be different from those who examine the patients, and all the drugs packages will be identified by unique numbers. Finally, the randomization table will be concealed from research staff by using closed envelops.

Compliance will be quantified by counting the number of capsules consumed during the interval between the two visits and presented as percentages (number consumed/number expected to be consumed) × 100). If compliance is less than 60 %, we will consider this case as noncompliant. Noncompliant patients will be included under the intention-to-treat analysis. Besides, at the analysis stage, we will compare intention-to-treat (if randomized, then we will analyze) and per-protocol analyses (those who have received the treatment compared to those who have not) and interpret the results.

### Outcome measures

The primary outcome in this study will be BP. Systolic and diastolic BPs will be measured by using a mercury sphygmomanometer twice on patient in a sitting position after a 10-minute rest and with a 15-minute interval. The measurement will be done every week during the intervention period. The mean systolic and diastolic BP will be computed. Mean arterial pressure will be calculated as systolic blood BP plus two times the diastolic BP divided by three [(SBP + 2 × DBP) ÷ 3]. The secondary outcome will include the biochemical analysis of plasma or serum for various biochemical and hematological markers. Table 1 provides a list of secondary outcome measures. Table 2 shows the flow chart of the study.

**Table 1:** List of biochemical markers

Outcome	Method of measurement
LFT (ALP, GGT, albumin, bilirubin)	Autoanalyzer (spectrophotometry)
Lipid profile (total cholesterol, HDL, LDL, TG)	Autoanalyzer (spectrophotometry)
RFT (serum creatinine, BUN)	Autoanalyzer (spectrophotometry)
Biochemical assessments(endothelin, TNF-α, NO, renin, angiotensin II, norepinephrine, malondialdehyde, urinary isoprostanes)	ELISA kits, spectrophotometry, gas chromatography/mass spectrometry
Hematological markers (PLT, HCT, PT, PTT)	Autoanalyzer assay kits
Fasting blood glucose	Autoanalyzer (spectrophotometry)

*LFT* liver function test, *ALP* alkaline phosphatase, *GGT* gamma-glutamyl transferase, *RFT* renal function test, *HDL* high-density lipoprotein, *LDL* low-density lipoprotein, *TG* triglycerides, *TNF-*α tumor necrosis factor-alpha, *NO* nitric oxide, *PLT* platelet, *HCT* hematocrit, *PT* prothrombin time, *PTT* partial thromboplastin time, *BUN* blood urea nitrogen

**Table 2:** Flow chart of the study

	1	2	3	4	5	6	Washout	7	8	9	10	11	12
Week	Screening	Baseline	1	2	3	4	(4 weeks)	8	9	10	11	12	16
In/exclusion criteria checking	×												
Enrolment	×												
Obtaining informed consent		×											
Physical examination	×												
Randomization	×	×											
Blood pressure	×	×	×	×	×	×		×	×	×	×	×	×
LFT	×	×				×		×				×	
RFT	×	×				×		×				×	
Lipid profile measurement		×				×		×				×	
Blood markers measurement		×				×		×				×	
Hematological indices and FBG measurement	×	×				×		×				×	
Compliance measurement			×	×	×	×		×	×	×	×	×	
Adverse effect checking			×	×	×	×		×	×	×	×	×	

*LFT* liver function test, *RFT* renal function test, *FBG* fasting blood glucose

### Sample size

The sample size for the study was calculated by using PASS 11 power and sample size software. Based on the software output, a two-sided *t* test will achieve approximately 80 % power when the total sample size of a 2 × 2 cross-over design is *n* = 44, the actual mean difference for systolic BP (as primary outcome) is 10, the square root of the within mean square error is 10, and the significance level is 0.05. Enrollment of 44 patients would provide an 80 % power to demonstrate a significant difference between the study arms. We expect approximately 10 % loss to follow-up. To achieve the calculated statistical power, we decided to recruit 50 patients in each strata (prehypertension and stage 1 hypertension group), equally allocated to sequences A or B. In total, 100 patients will participate in this trial. To minimize loss to follow-up, we give the participants a visit card. The next visit time is recorded on the card based on the study protocol. In addition, we call the participants, the day before, to remind them of their visit time, and again on the day after if they fail to attend their visit on time to re-invite them to reschedule soon.

### Adverse effects

No reports exist of serious adverse effects in any of the previous human studies with the 1-g resveratrol treatment, including a recent study from our group where we used a similar dosage of resveratrol in diabetic participants [11]. Nevertheless, any adverse event during the study will be recorded. If the adverse effect is serious enough to require medical attention, it will be reported as soon as possible, and the Data and Safety Monitoring Board (DSMB) will make a decision on whether or not blinding should be removed and whether the patient should be excluded from the study. In the case of a fatal or severe event requiring hospital admission, reporting should occur on the same day to the principal investigator. For any adverse effect in its early stages (before any link with the intervention is established) necessary treatment will be given. The DSMB will be responsible for studying each case individually to verify a possible link to study products. The principal investigator will be responsible for managing the adverse effects clinically. Necessary services will be provided free of charge. If any unexpected serious or fatal adverse effect occurred that is believed to be due to the consumption of resveratrol, the trial will be terminated. The decision will be made by the DSMB.

### Data quality control and management

All staff members who will collect and handle the data are well trained for managing clinical data. Adequate attention will be given to collect accurate and valid data and set up a regular monitoring scheme by qualified staff. Original hard copies of patient records will be kept at the recruitment center, a copy will be sent to the research deputy of BPUMS, and the data will be available only to designated researchers involved in the trial. All patient documents that are sent or received will be stored after taking into consideration safety and security issues.

Necessary schemes will be set up to control the quality of drug delivery, storage and handling, clinical examinations, and laboratory tests.

### Physical examination

Anthropometric parameters and clinical characteristics of the participants, including age, sex, height, weight, and body mass index (BMI) will be measured. The participants will also be asked to fill out a standard questionnaire form (developed by the United States Department of Agriculture) regarding their typical food intake, including the amount of salt, alcohol, green tea, coffee, grapes, peanuts, wine, berries, and lifestyle (exercise, smoking, sleeping habits, and rest) [15, 18]. In addition, the amount of vitamins and other micronutrients supplemented to the diet will be included. At baseline, the patients will be asked to fast (10-hour to 12-hour overnight fast) for blood collection. The blood samples will be collected before the first stage of the study, after the 1-month intervention, after the 1-month washout, and at the end of the study. Then, the serum will be separated and given a code number and stored at −80 °C until analysis.

### Statistical analysis

Data will be analyzed on an intention-to-treat basis, defined as all randomized patients who received at least one dose of study medication. Patients with no data recorded for a parameter will be excluded from the analysis of that particular parameter. The statistician will remain blinded to the status of the patients with regard to the intervention. Data will be analyzed stratified by prehypertension or stage 1-hypertension groups.

Final analyses will be conducted after the trial is finished or a decision has been made to stop the trial by scientific steering committee, which may occur after the interim analysis if the members are satisfied with the strength of the evidence.

Because of a relatively long washout period, we do not expect a significant carryover effect. However, we will check for a carryover effect by using a two-group independent *t* test to compare the average effects of outcomes for the two sequences. Treatment, sequence, and period effects will be estimated by using an analysis of variance model for all outcome variables. Baseline values of outcome variables as well as potential confounding factors will be controlled by using repeated measure analysis of variance models. Appropriate post-hoc analysis using Bonferroni correction for multiple comparisons will be performed.

Data will be analyzed by using the pk crossover menu of Stat/SE 11.0 statistical software.

## Discussion

The increasing need for alternative strategies for controlling hypertension can be addressed by identifying promising nutraceutical candidates. In this regard, resveratrol has demonstrated great potential in preventing and/or reversing cardiovascular diseases including hypertension in preclinical studies. Recent meta-analyses [15, 18] that reviewed successful clinical trials [11, 12] concluded that resveratrol may be considered as an adjuvant therapeutic candidate for managing type 2 diabetes. In light of these promising clinical findings and previously reported preclinical evidence, resveratrol appears to be a potential antihypertensive agent. The efficacy of resveratrol has yet to be clinically investigated in patients with hypertension. This trial will be the first study to investigate the potential of therapeutic use of resveratrol for the management of BP in patients diagnosed with prehypertension and type 1 hypertension. The outcomes from this study will help to determine the efficacy of short-term resveratrol treatment in hypertensive patients and bridge the gap with regards to the current preclinical and clinical evidence. This study will also help in collecting further information with regard to identifying a therapeutically effective dose of resveratrol in controlling cardiovascular disease risk factors. Importantly, if a positive outcome is identified, it will provide us with an effective therapeutic strategy to combat hypertension and would pave the way for achieving enormous public health benefit.

### Study limitations

This trial is designed as a pilot study to investigate the efficacy of resveratrol as a blood-pressure-lowering agent in a specific population. In addition, the trial has a small sample size. Hypertension is a condition that requires long-term medication. Because the trial duration is only 4 weeks, longer-term studies need to be conducted to evaluate the efficacy of resveratrol as a sustained treatment option. In this study, participants will receive daily only a single, high dose of resveratrol, which will be taken twice throughout the study (a high dose that is well tolerated). Further trials may be needed to ascertain whether resveratrol can lower BP at a much lower daily dose. Another limitation is that a pharmacokinetics study will not be done in this trial to understand the absorption, distribution, metabolism, and excretion of resveratrol in hypertensive patients; pharmacokinetics may have helped to draw a correlation between the plasma bioavailability and the actual physiological effect. In this trial, stage 1-hypertensive patients will be on standard therapy for hypertension as well; therefore, it may not be possible to understand the standalone efficacy of resveratrol in lowering BP in these patients. Extensive toxicological analysis will also not be conducted as part of this study.

### Trial status

Recruitment is in progress.

## Abbreviations

ALP, alkaline phosphatase; Ang, angiotensin; BMI, body mass index; BP, blood pressure; BUN, blood urea nitrogen; DSMB, Data and Safety Monitoring Board; GGT, gamma-glutamyl transferase; HCT, hematocrit; HDL, high-density lipoprotein cholesterol; LDL, low-density lipoprotein; LFT, liver function test; NO, nitric oxide; PLT, platelet; PT, prothrombin time; PTT, partial thromboplastin time; RLT, renal function test; SHR, spontaneously hypertensive rat; TG, triglyceride; TNF-α, tumor necrosis factor- α

## Appendix 1

### Informed consent

The informed consent process and form will consist of the following points:

- The patients will be informed that they are participating in a clinical trial that assesses the effectiveness of oral resveratrol in prehypertension and stage 1 hypertension. Previous studies have shown that the drug does not have serious adverse effects at levels prescribed in this study and has had favorable effects on the manifestations of the disease.
- This clinical trial will be done on 50 patients with prehypertension (diastolic BP and systolic BP, 80–89 mmHg and 120–139 mmHg, respectively) and 50 patients with stage 1 hypertension (diastolic and systolic, 90–99 mmHg and 140–159 mmHg, respectively) in the School of Medicine of Bushehr University of Medical Sciences in Iran.
- Participants do not have to make a prompt decision, and they can consult anybody they want before making their final decision.
- All the participants should enter and stay in the study voluntarily, and with full knowledge of the situation. Therefore, they can leave the study at any time, and this will not affect their ordinary appointments with their doctor and they will receive their treatment as before.
- All participants will undergo a thorough investigation including clinical examinations and laboratory tests to find out whether or not they are eligible to enter the study. Half of the participants from both groups (25 patients each) in this study will receive resveratrol capsules (500 mg twice a day) generously supplied by Biotivia Longevity Bioceuticals LLC Company, USA, in the first period. The second half of the participants both groups (another 25 patients) will receive a capsule identical to resveratrol but with no active ingredient (produced by Biotivia Longevity Bioceuticals LLC Company, USA). After the completion of the 4 weeks, the participants will crossover to receive the active drug or placebo for another 4 weeks.
- During the treatment, participants will be examined regularly (every week) by a physician or cardiologist and will be consulted routinely. The BP and adverse effects of the treatment will be assessed in each visit and will be continued for 1 month after the treatment period is over.
- In the event of adverse effects in participants during the study and up to 1 month after the study ends, all expenses for the necessary treatment and laboratory investigation will be paid for by the chief investigator as a part of the grant.
- All information gathered during this study will be kept confidential and will only be used by authorized staff for analysis. The results from this study will be published in scientific journals for public use.
- Expenses endured by participants because of this study, including travel expenses to come to the clinic, will be reimbursed by the investigation team during the study period.
- Points of contact will be provided for participating subjects to address their general and scientific enquiries. Furthermore, a contact point to receive complaints will be introduced.
- Participants are not waiving any of their legal rights by signing this consent form, or releasing the investigator from their legal and professional responsibilities.
- We will also let participants know about any new information that may affect their health, welfare, or willingness to stay in this study.
- Participants are free to ask any questions they may have about their treatment and their rights as a research participant. If any questions come up during or after the study or if they have a research-related injury, they can contact the study coordinator.
- Participants are encouraged not to sign this consent form unless they have had a chance to ask questions and have received satisfactory answers to all of their questions
